# Lack of children in public medical imaging data points to growing age bias in biomedical AI

**DOI:** 10.21203/rs.3.rs-7713694/v1

**Published:** 2025-10-03

**Authors:** Lauren Erdman, Nicholas Heller, Ping He, Alexander Towbin, Irene Chen, Alex Lu, Stanley Bryan Hua

**Affiliations:** Cincinnati Children’s Hospital Medical Center; Cleveland Clinic; The Hospital for Sick Children; Cincinnati Children’s Hospital Medical Center; Berkeley; Microsoft Research; University of California, Berkeley

## Abstract

**Background.:**

Artificial intelligence (AI) is transforming healthcare, but its benefits have not been equitably distributed, with children being particularly overlooked. Only 17% of FDA-approved medical AI devices are labeled for pediatric use. We hypothesized that this disparity may be due to a fundamental data gap in pediatric medical imaging.

**Methods.:**

To test this hypothesis, we performed a systematic review of 180 publicly available medical imaging datasets to assess pediatric data representation. To identify the primary data sources used for methods development, we first surveyed papers from a machine learning imaging conference. Finally, we evaluated the performance of adult-trained chest radiograph models when applied to pediatric populations to quantify potential age-related bias.

**Results.:**

Our systematic review found that children represent less than 1% of the data in public medical imaging datasets. The majority of machine learning conference papers we surveyed relied on publicly available data for model development. Furthermore, we found that adult-trained chest radiograph models exhibit significant age bias when applied to pediatric populations, with higher false positive rates in younger children.

**Discussion.:**

This study highlights the urgent need for increased pediatric representation in publicly accessible medical datasets. Our findings suggest that the lack of pediatric data may contribute to the scarcity of AI tools for children and the poor performance of adult-trained models in this population. We provide actionable recommendations for researchers, policymakers, and data curators to address this age equity gap and mitigate the potential harms of AI systems not trained on pediatric patients.

## Summary

Our analysis reveals a critical healthcare age disparity: children represent less than 1% of public medical imaging datasets. The lack of pediatric representation leads to downstream biases in machine learning models, with the youngest patients facing the highest risk of model failure.

## Background

A growing number of studies highlight the potential for artificial intelligence (AI) to improve patient care^1,2^. A prerequisite for medical AI is the availability of high-quality datasets such as MIMIC-III^3,4^ and CheXpert^5^. The public release of these datasets attracts efforts from the machine learning community to develop new state-of-the-art algorithms, leading to advancements in medical AI, including but not limited to sepsis prediction, acute critical illness management, and insights for chronic diseases^6–9^.

In the United States, growing interest and development in medical AI is reflected in a year-on-year increase in the number of AI-enabled medical devices approved by the Food and Drug Administration (FDA). Yet, studies into FDA approvals reveal that fewer than 1 out of 5 devices are approved for pediatric use^10^, and ages of study participants are not reported in 81.6% of devices^11^. The neglect of age-related differences mirrors historical patterns in drug^12^ and device development^13,14^, where children suffer disparities in quality of care compared to adults^15–17^.

A growing concern in medical AI is the presence of biases in algorithmic output, driven by missing data and a history of inadequate care for the vulnerable populations these tools aim to help^18^. In clinical care, these algorithmic biases have the potential to harm patients, especially in high-risk applications like disease diagnosis and management. While algorithmic biases have been extensively studied in the context of sex and race^18^, recent studies indicate that age representation is equally important, as AI trained on adult data display age-related biases against children^19–23^. Underlying the challenging transfer of adult AI to children are fundamental differences in anatomy and physiology^24–27^, in addition to clinical differences in pediatric disease risk, presentation and management^28–35^. These stark differences suggest that pediatric data is critical in developing AI applications for children.

While the availability of public data is beneficial for developing medical AI, motivations for using public datasets vary greatly from researcher to researcher. In communities focused on a particular application, efforts tend to concentrate on a few key benchmark datasets^36,37^. In contrast, challenges attract generalist machine learning practitioners to work on novel applications. Despite the ecosystem of public datasets and challenges that help drive AI development, pediatric representation in these crucial resources and its implications remain largely unknown. The purpose of this study is to shed light on pediatric representation in public medical imaging datasets and concretely show the potential harm of pediatric underrepresentation in deploying machine learning models trained on adults for pediatric use.

## Methods

### Analyzing Data Use in Recent AI for Medical Imaging Papers

Machine learning practitioners leverage available datasets to help drive advancements for medical AI, and conferences serve as their primary publication venue. To understand patterns of dataset usage in medical AI research among machine learning practitioners, we surveyed 2023 and 2024 papers accepted to a conference, focused on cutting-edge deep learning methods for medical image analysis. This led us to review papers from the conference *Medical Imaging with Deep Learning* (MIDL)^38^. Papers are filtered for those that use structural non-invasive internal imaging data, further defined in the dataset inclusion criteria. For each paper, we recorded the dataset(s) used, and then for all datasets, we identified how the dataset was made available. Based upon this, we identified sources for datasets: datasets collections, challenges and benchmarks. More details are provided in the Dataset Selection section.

### Review of Public Medical Imaging Data

**Figure 1:**
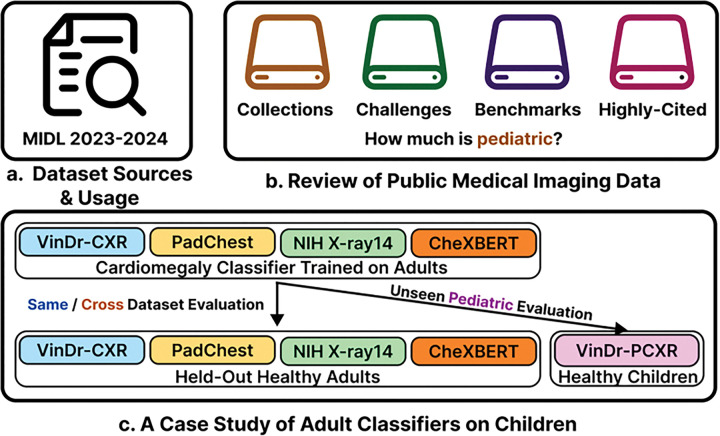
Overview of methodology. **(a) Analyzing Data Use in Recent AI for Medical Imaging Papers.** Medical Imaging with Deep Learning (MIDL) papers are reviewed to scope public dataset sources and usage and presence of pediatric applications of AI for medical imaging. **(b) Review of Public Medical Imaging Data.** Public medical imaging datasets are taken from multiple sources and reviewed for pediatric representation. **(c) A Case Study of Adult Cardiomegaly Classifiers on Children.** Cardiomegaly classifiers are trained on adult chest radiograph datasets and evaluated on held-out healthy adults and healthy children.

Given the sources of public datasets identified, we conducted a systematic review of publicly available medical imaging datasets by collecting datasets from each data source to assess pediatric representation in public data, (Figure 1a, 1b). We account for bias from adult over-representation in datasets with cancer patients by assessing pediatric representation in non-cancer datasets.

#### Dataset Inclusion Criteria

To be eligible for our systematic review, a dataset must meet the following conditions:

##### 1. Structural Non-Invasive Internal Imaging.

The dataset must contain data for medical imaging modalities that capture internal anatomical structures of organs and tissues and don’t require breaking the skin or physically entering the body. This strictly includes fundus imaging, ultrasound, magnetic resonance imaging (MRI), X-ray, computed tomography (CT), or their common variants (e.g., mammography, retinal OCT). Fluoroscopy and nuclear imaging were excluded, as little to no public datasets were found exclusively with these modalities.

##### 2. Accessibility.

The dataset must be publicly downloadable and usable without requiring manual authorization from the data provider.

To avoid redundancy in downstream analyses, we excluded datasets that simply repurpose datasets already annotated. For datasets that are updated annually such as the Brain Tumor Segmentation challenge (BraTS)^39^, only the latest versions (as of January 1, 2025) containing complete and original data are included. If a dataset contains both data that falls within the inclusion criteria and data that falls outside the inclusion criteria, only the subset of data that falls within the inclusion criteria is annotated and included in subsequent analyses.

#### Dataset Annotation

For datasets meeting the inclusion criteria, we categorized how patient ages are reported and attempt to identify the proportion of pediatric patients -- patients aged 17 or younger in each dataset. We identified the following patient age reporting strategies: *patient-level*, *binned patient-level*, *summary statistics*, *task/data description* and *not documented*. In the best case, metadata files are provided containing ages for each study participant, which allows for computing the exact proportion of pediatric patients in a dataset. In *binned patient-level* reporting, patient ages are provided in age bins (e.g., 20–30 years old). If we could not infer the presence and proportion of pediatric data through available metadata files, we scanned associated publications for *summary statistics* related to patient ages. If none of the above methods were successful, we attempted to determine if there was no pediatric presence by scanning *task or data descriptions* for any clear mention or indicators of “adult-only” data in the associated publication, dataset’s official website or portal. Otherwise, a dataset was labeled as *not documenting* age.

#### Dataset Selection

To curate datasets for our systematic review, we collected and organized them by their source.

*Dataset Collections*refer to repositories curated for diverse research and practical applications, including medical imaging. For this study, we focused on clinical data repositories containing medical imaging datasets, either derived from clinical studies or explicitly released for developing machine learning algorithms. Our analysis included the following dataset collections: **(a)** The Cancer Imaging Archive (TCIA)^40^, **(b)** Stanford AI in Medicine & Imaging (AIMI)^41^, **(c)** UK BioBank^42^, **(d)** Medical Imaging and Data Resource Center (MIDRC)^43^, and **(e)** OpenNeuro^44^. For TCIA and AIMI, we focused on complete datasets strictly with patient-specific age metadata, while for UK BioBank, MIDRC and OpenNeuro, we relied on aggregated patient statistics provided by their respective portals.

*Challenges*play a pivotal role in advancing state-of-the-art machine learning, often targeting novel or domain-specific applications. Machine learning challenges for medical imaging are commonly hosted on platforms such as the Grand Challenge^45^, Kaggle^46^ and the Radiological Society of North America (RSNA)^47^ in conjunction with conferences. For this study, we annotated datasets from challenges hosted on the listed platforms, launched in the last five years (2019 to 2024).

*Benchmarks*consist of one or more pre-existing or novel datasets designed to evaluate and compare algorithm performance on specific and often well-established tasks. While numerous datasets can serve as benchmarks, we focused on three explicitly labeled as such: two benchmarks for multi-organ segmentation:Medical Segmentation Decathlon (MSD)^48^ and AMOS^49^, and one benchmark for assessing fairness in medical imaging machine learning: MedFAIR^50^.

*Highly-Cited Datasets* serve as a means to identify datasets that don’t belong to a distinct dataset collection, challenge or benchmark, yet have become foundational resources in the academic community, serving as the basis for numerous significant contributions over time. To identify these datasets, we leveraged Meta’s PapersWithCode^51^, which ranks imaging datasets by citation count. We selected and annotated the top 30 datasets meeting our inclusion criteria. When aggregating datasets across dataset sources, we remove any duplicate datasets found via PapersWithCode and the other dataset sources. In analysis on highly-cited datasets alone, all 30 identified datasets are used.

#### Data Breakdown by Modality and Task

To investigate if pediatric representation differs by application, we first filtered for datasets where the proportion of pediatric data can be estimated, then we categorized datasets by imaging modalities and machine learning tasks. Specifically, machine learning tasks are classified under: a) *condition classification* (e.g., pneumonia classification), b) *anatomy segmentation* (e.g., organ or tissue segmentation), c) *lesion segmentation* (e.g., tumor segmentation), d) *disease risk segmentation* (e.g., fractured rib segmentation, organs-at-risk segmentation), and e) *image enhancement* (e.g., MRI image reconstruction, synthetic image generation).To perform our analysis, we aggregated the number of medical images across datasets by imaging modality and task. As any one dataset can contain data from multiple imaging modalities, we attempted to identify what proportion of the dataset comprises each modality, and if not possible, we assumed the images are split evenly across modalities.

#### Dataset Repackaging

In our review, we observed cases in which a released dataset includes data previously released in a pre-existing public dataset. We term this phenomenon “dataset repackaging”. We identified all datasets that perform dataset repackaging. On those datasets, we annotated the original datasets they reference and extracted age-related metadata from the original dataset. As many foundation models are often developed on public datasets, we supplemented our analysis by considering patient ages in the datasets used by MedSAM, a medical foundation model.

### A Case Study of Adult Cardiomegaly Classifiers on Children

To examine how medical AI developed on adult data may underperform on children, we designed a case study, which leverages a well-established anatomical difference between adults and children. Children have larger hearts relative to their chest, compared to adults^52,53^. Cardiomegaly is the clinical description of an abnormally large heart, and to account for pediatric differences, the thresholds on acceptable cardiothoracic ratios are higher for children^53^. We trained models to classify cardiomegaly from adult chest radiographs and evaluated these models on children (Figure 1c).

#### Data.

For our experiments, we utilized five large publicly available chest x-ray datasets: **(a)** VinDr-PCXR^54^, **(b)** VinDr-CXR^55^, **(c)** NIH X-ray14^56^, **(d)** PadChest^57^ and **(e)** CheXBERT^5,58^. VinDr-PCXR is a dataset of posteroanterior (PA) view chest x-rays from children aged 0 to 10 years, collected at a major pediatric hospital in Vietnam. Healthy children with age annotations in the VinDr-PCXR dataset are used for held-out pediatric evaluation. The remaining chest x-ray datasets consist primarily of adult data and are used for training, calibration and held-out evaluation on healthy adults. The VinDr-CXR data is from two hospitals in Hanoi, Vietnam, while PadChest data is from a hospital in Barcelona, Spain. Meanwhile, the NIH X-ray14 and CheXBERT are from hospitals in the United States associated with NIH and Stanford, respectively. NIH X-ray14 is the extended version of the original NIH ChestX-ray8 dataset, while CheXBERT is the CheXpert dataset with CheXBERT-assigned labels.

#### Model.

A ConvNeXt-B (89M parameters) is chosen as our base neural network architecture^59^. For each of the datasets, a network is trained for at most 50 epochs using the AdamW^60^ optimizer with a learning rate of 0.0001 to optimize the binary cross-entropy loss. During each training step, 32 random x-ray images are sampled equally from both classes, and MixUp is used for data augmentation^61^. Parameters from the epoch with the best validation set loss are kept.

#### Evaluation.

The probability threshold (or operating point) for each classification network is adjusted to maximize Youden’s J statistic (defined as sensitivity + specificity − 1) on their corresponding calibration set. Each adjusted model is then used to predict cardiomegaly on the healthy children in the VinDr-PCXR dataset and healthy adults in each of the adult datasets. The proportion of false positives is reported at the dataset level and stratified by age groups. To determine statistically significant differences, bias-corrected and accelerated bootstrap is used to construct 95% confidence intervals around the false positive rate^62^. To identify patterns between age in years and false positive rate, we normalized the false positive rates by each model and computed a Pearson r correlation.

## Results

### MIDL studies frequently use public data with little pediatric applications.

Majority (37/46; 80%) of MIDL 2023–2024 studies utilize public datasets. Among these studies, only one (2.2%) directly applied its work to pediatrics. Moreover, 85% (39/46) of studies and 35% (22/62) of cited public datasets failed to report patient ages altogether – a fundamental metadata gap that obscures representational analysis. Only one of the 46 studies used a dataset containing primarily pediatric data, and while 9 additional studies used datasets containing pediatric data, less than 5% of the patients in those datasets were children. From these findings, we hypothesized that limited pediatric applications may stem from a lack of public pediatric datasets, motivating a larger review of the presence of pediatric patients in public medical imaging data.

### Children are underrepresented in public medical imaging data.

**Figure 2:**
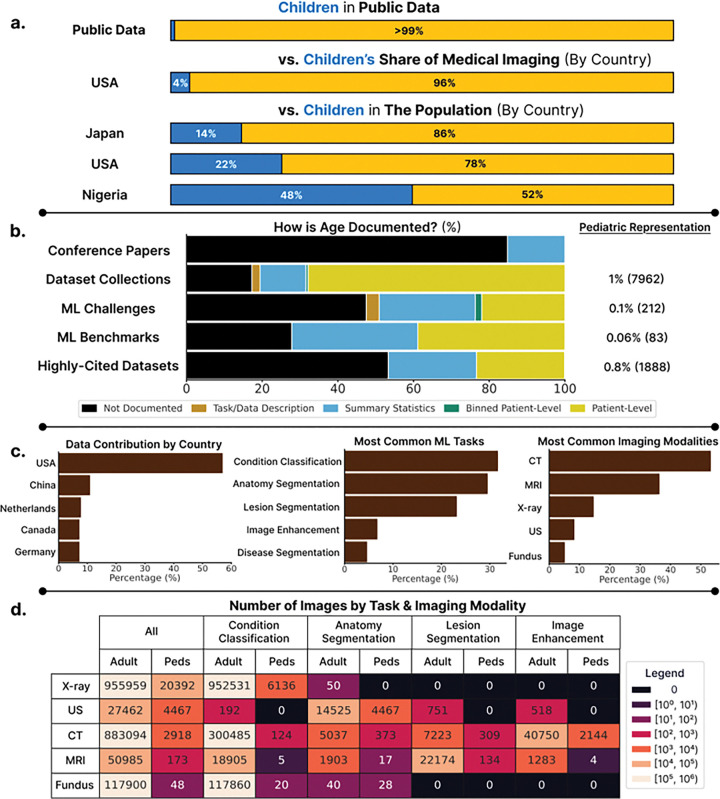
**(a) Children make up less than 1% of patients in public medical imaging datasets**. This is contrasted with the percentage of medical imaging examinations that were pediatric in 2016 performed in the United States^63^, and population percentages provided by the United Nations^71^ in 3 countries selected from our dataset review. **(b) There is a lack of standardized patient age reporting in datasets released and the papers that cite them**. For each dataset source, the percentage and number of pediatric patients is reported across datasets where age is documented (right). **(c) Descriptive plots on source countries, tasks and imaging modalities in reviewed public medical imaging datasets**. Percentages provided describe the percentage of datasets that satisfies the condition. **(d) Stratifying the number of images by modality and task across datasets reveals data scarcity for pediatric applications**.

Our comprehensive review of 180 public datasets reveals a large disparity: only 3.3% (6/180)of datasets are pediatric-only, while an additional 13.9% (25/180) of datasets contain both adult and pediatric data. Among 117 datasets with sufficient patient age information, children represent under 1% of patients in public medical imaging datasets(4558 of 489,743). In contrast, pediatrics composed 4% of all imaging examinations performed across 7 healthcare systems in the United States^63^, where most of these patients were imaged (Figure 2a). This underrepresentation persisted across all dataset sources (Figure 2b, Table 1). In major collections like MIDRC^64^, TCIA^40^, and Stanford AIMI^65^, children comprised merely 1–2% patients. Other data collections such as the UK BioBank^42^ exclude pediatric patients by design. Among 59 ML challenges between 2017–2024, only two explicitly targeted pediatric applications. Even established benchmarks showed minimal pediatric presence: zero evidence of pediatric data in MSD^48^, and less than 1.1% in AMOS^49^ and 0.1% in MedFAIR^50^. Among the 30 highly-cited datasets, children represented just 0.8% of patients. To account for potential adult over-representation in datasets with cancer patients, we revisit pediatric representation excluding such datasets, removing more than half of the datasets (95 of 180), and this leads to children representing 1.1% of patients (4421 of 387,691).

The true extent to which children are underrepresented is obscured by the lack of standardized age reporting across datasets. This led to the exclusion of 63% of highly-cited datasets, 83% of benchmark datasets, 51% of ML challenges and 54% of datasets in OpenNeuro, TCIA and Stanford AIMI collections.

### Stratifying by modality and task reveal many applications for which virtually no pediatric data exists.

Grouping adult and pediatric images across datasets, we found little to no pediatric representation in many modality-specific tasks relative to adults (Figure 2d). When comparing by imaging modality alone, the data gap varies greatly with the smallest in ultrasound: an estimated 6 adult for every 1 pediatric ultrasound image, while larger gaps are observed for X-ray (47:1), MRI (295:1), CT (302:1) and fundus (2456:1). In our analyses, often one dataset comprises the majority of pediatric data for each modality: X-ray (70% from RSNA Pediatric Bone Age^66^), ultrasound (100% from EchoNet-Peds^20^), CT (72% from LoDoPaB-CT^67^), MRI (77% from BONBID-HIE^68^) and fundus (100% from CHASE DB 1^69^).

### Patient ages are often discarded and overlooked in data repackaging efforts.

Nearly 10% (16/180) of the datasets we reviewed repackage data from other public datasets. Among datasets that repackage data, half (8/16) used public datasets that included age metadata. Yet, patient ages were discarded in the resulting datasets. Further, little attention is paid to patient ages in recent efforts to combine public datasets for foundation model development. In MedSAM, 26 public datasets are aggregated to create a dataset of nearly 1.6 million medical image-mask pairs with no information on the distribution of patient ages. Pediatric data is only present in 3 of the 26 datasets with an estimated 14 children among 8508 patients overall. For recurring competitions, pediatrics appears to be a more recent and intentional development. While BraTS^39^ exclusively targeted adults from 2012 to 2022, BraTS 2023^22^ was the first to focus on pediatric gliomas. While this indicates a shift towards recognizing the importance of pediatrics, newer pediatric datasets currently represent a small fraction over a legacy of adult-focused datasets. These observations suggest that data repackaging efforts, if not intentional about age, may lead to age-imbalanced datasets given the current data landscape.

### Pediatric data is necessary to develop pediatric-targeted AI applications as adult models are not guaranteed to transfer well to children.

**Figure 3:**
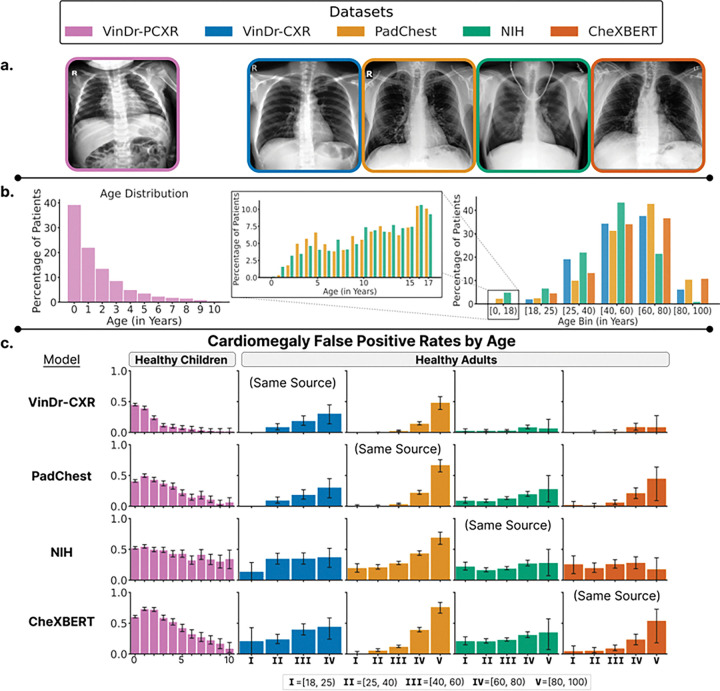
**(a) Example chest x-ray image** from a healthy patient in each of the chest x-ray datasets; the pediatric evaluation dataset VinDr-PCXR (**left**) and the adult-majority training datasets (**right**). **(b) Distribution of patient ages** in VinDr-PCXR dataset (**left**) and in the adult-majority datasets (**right**). For adult-majority datasets with pediatric data (i.e., Padchest, NIH), the pediatric age distribution is skewed towards older children with almost no infants at all (**middle**). **(c) Cardiomegaly classifiers trained on adult data increasingly mispredict cardiomegaly on younger children**. The y-axis represents the false positive rate, and error bars display bootstrapped 95% confidence intervals.

In the absence of tailored solutions, medical devices and drugs designed for adults may be used on children^15,16^. The untested pediatric use of medical devices and drugs introduces unknown risks to children^17,70^, and we argue similar risks exist in the new era of AI-enabled medical devices. To demonstrate hidden age biases in adult-trained AI models, we trained cardiomegaly classifiers on four adult chest X-ray datasets, and measured their false positive rate on held-out healthy adults and on healthy children from a held-out pediatric X-ray dataset (Figure 3). On healthy adults, the models did not display consistent age-related bias across adult datasets. In children, however, we found a significant age-related bias: for patients under 11 years old, the rate of false positives increased for younger patients (Pearson r=−0.928, p-value<0.0001), irrespective of the dataset the model was trained on. Additionally, we showed that correcting for differences in image contrast using histogram matching fails to rescue the high false positive rates for younger children across models **(Supplementary Figure 2)**.

## Discussion

Our study reveals a critical data gap: children represent less than 1% of all patients in public medical imaging data. Only 1 of the 30 (3.3%) most cited medical imaging datasets is pediatric, implying little attention to pediatric AI historically. Only 2 in 59 (3.4%) medical imaging challenges between 2017–24 are pediatric, and only 1 of 46 (2.1%) MIDL studies in 2023–24 targets pediatrics. Altogether, these findings suggest a lack of pediatric AI applications being actively worked on in the research community, potentially driven by the pediatric data gap. In the absence of pediatric AI models, practitioners may opt for the off-label use of adult AI models. While prior work has shown that adult-trained AI algorithms can underperform on children^19–23^, our case study recapitulates a known difference between adults and children. Children display larger heart-to-chest size ratios^52,53^, and adult-trained models fail to account for this age-related difference, increasingly mispredicting cardiomegaly in young healthy children.

To identify and prevent age-related model failure in practice, pediatric representation is crucial yet currently overlooked in publicly available medical imaging data. Beyond representation, we also identify issues with documentation: most public datasets fail to report patient ages, which limits age-related analyses and model corrections for age-related bias. Our analyses further establish that pediatric representation in public data is not only small but fragmented. Pediatric data in each modality is dominated by 1 dataset, and many tasks see virtually no pediatric representation in certain modalities. With growing demand for larger datasets to fuel foundation model development, our findings suggest that the lack of diversity of pediatric datasets may bias dataset curators towards aggregating adult datasets, and the lack of age metadata may prevent attempts to balance data by age.

Additionally, the pediatric data gap varies by imaging modality. We observe 1 pediatric image for every 6 adult images in ultrasound, compared to CT (1 for 302) and MRI (1 for 295). In practice, children do receive more ultrasounds than CT and MRI compared to adults^63,72,73^. One explanation is older adults receive more frequent cancer screening, requiring CTs and MRIs. Another reason is a preference to reduce exposure to harmful radiation for children^74^, and this often comes at the cost of reduced image contrast in radiographs^75,76^. In addition to adjusting for patient sizes and physiology, pediatric imaging presents unique challenges like managing patient anxiety and movement to avoid motion artifacts. This requires gaining a child’s trust and providing emotional support^23,77^. Altogether, these differences in medical practice are strong contributors to the differences in types of images acquired and their characteristics, compared to adults. While scaling public pediatric data is necessary to resolve the data gap, we caution against data collection efforts that may attempt to standardize imaging practices to match that of adults, as it poorly reflects pediatric imaging in practice.

A key challenge in addressing the pediatric data gap is establishing a clear end-goal. One study found that children accounted for 4% of all imaging examinations in 2016 from data across 7 healthcare systems in the United States^63^. While pediatric representation of 4% may be seen as a reasonable target, the true end-goal is to develop safe pediatric AI tools. Achieving safe pediatric AI necessitates iterative algorithm design and validation, and access to sufficient pediatric data, which is simply not available in the present.

We acknowledge the existence of systemic barriers that discourage pediatric data curation and release. Developing medical devices and drugs for pediatric use faces unique economic and regulatory hurdles compared to medical devices and drugs for adults: the rarity of some pediatric pathologies lead to small sample sizes, poor patient recruitment and high development costs^78,79^ and pediatric clinical trials requiring greater standards for consent, safety and efficacy^79–82^. While changes in public policy have greatly encouraged pediatric drug and device development, similar policy changes have yet to reach medical AI and encourage pediatric data collection^83–85^. An encouraging sign is the formation of new initiatives to support pediatric AI equity^86,87^. However, we believe that closing the gap will require collective efforts from diverse stakeholders.

### A solution to the pediatric data gap will not arise naturally.

We urge the broader ML for health community to take action. For healthcare institutions and researchers, we encourage greater collaboration and initiatives to collect, prepare and release AI-ready pediatric data to the public; to support the development of pediatric AI applications. We emphasize the necessity of age representation from infancy to adulthood and the reporting of ages at the patient-level. Greater age representation and transparency will help to identify age ranges where AI models are most effective, leading to improved safety beyond pediatric cases. Building on public pediatric data releases, we encourage the creation of challenges and benchmarks to leverage the benefits of community-driven algorithm development and spread awareness on the necessity for pediatric AI. For policy-makers, we hope for policy changes that lower barriers to and encourage pediatric data collection and sharing. In addition, we argue for greater scrutiny in the pediatric evaluation of AI applications, focusing on precision in targeted age groups and sufficient representation across ages of human development.

### Limitations

We acknowledge two limitations of our study. First, there may exist pediatric datasets that were not captured in our systematic dataset search, particularly in data platforms without centralized curation such as Zenodo and Harvard Dataverse. Our analysis, however, captures key pathways in which datasets may be found by ML practitioners. Another contributing factor is stricter access controls for pediatric datasets, which ultimately led to the exclusion of most pediatric cancer datasets in TCIA. Lastly, poor age reporting among public datasets led to the exclusion of many datasets in our analysis. Although pediatric data may exist among the excluded datasets, our findings suggest they may only represent a small portion.

## Conclusion

Despite forming a significant portion of the population, children are almost invisible in the datasets driving advancements in AI for medical imaging, constituting less than 1% of public medical imaging datasets.The glaring underrepresentation of pediatric patients and the poor reporting of patient ages in public medical datasets are critical oversights that limit the development of safe AI for pediatric use. This work not only quantifies the extent of this problem but demonstrates the potential risks of using adult-only AI models on children. Lastly, we provide recommendations to encourage the community to take action now in addressing the pediatric data gap.

## Supplementary Material

Supplementary Files

This is a list of supplementary files associated with this preprint. Click to download.

• SupplementaryMethods.docx

## Figures and Tables

**Table 1.** Summary statistics of public medical imaging datasets studied from each data source.

	N	Countries	Institutions	Datasets	Patients
**Dataset Collections**	5	13	68	958	788.9K
**ML Challenges**	59	28	103	59	214.3K
**ML Benchmarks**	3	14	39	18	143.1K
**Highly-Cited Datasets**	30	15	41	30	259.3K

Statistics are estimated using papers and metadata associated with each dataset. **N** denotes the number of collections, challenges, benchmarks or highly-cited datasets. The remaining columns provide counts on the number of unique countries, unique institutions, unique datasets and estimated number of patients.

## Data Availability

All data and code to reproduce analyses in this paper are available on GitHub at https://github.com/stan-hua/ped_vs_adults-cxr. All annotations for our dataset review are provided in the attached **Supplementary File 1**. The datasets used in this cardiomegaly case study are publicly available and subject to their data use agreements. The MIMIC-CXR, VinDr-CXR, and VinDr-PCXR datasets were downloaded from PhysioNet. Meanwhile, PadChest, NIH and CheXBERT were downloaded from their respective sources. We provide detailed instructions on how to prepare the data on GitHub at https://github.com/stan-hua/ped_vs_adults-cxr/tree/main/data.
